# Structure of Patients’ Temperament Traits as a Risk Factor for Anxiety and Depression in Patients with Asthma and Chronic Obstructive Pulmonary Disease (COPD)

**DOI:** 10.3390/jcm14103414

**Published:** 2025-05-13

**Authors:** Paula Zdanowicz, Zbigniew Włodzimierz Pasieka, Radosław Wujcik, Piotr Jarosław Kamola, Adam Jerzy Białas, Tadeusz Pietras

**Affiliations:** 1 Department of Biomedicine and Experimental Surgery, Medical University of Lodz, 90-419 Lodz, Poland; zbigniew.pasieka@umed.lodz.pl; 2 Faculty of Psychology, SWPS University of Social Sciences and Humanities, 03-815 Warsaw, Poland; rwujcik@swps.edu.pl; 3 Department of Haemostasis and Haemostatic Disorders, Medical University of Lodz, 90-419 Lodz, Poland; piotr.kamola@umed.lodz.pl; 4 Department of Pulmonary Rehabilitation, Regional Medical Center for Lung Diseases and Rehabilitation, Blessed Rafal Chylinski Memorial Hospital for Lung Diseases, Medical University of Lodz, 91-520 Lodz, Poland; adam.bialas@umed.lodz.pl; 5 Department of Clinical Pharmacology, Medical University of Lodz, 90-153 Lodz, Poland; taduesz.pietras@umed.lodz.pl

**Keywords:** asthma, chronic obstructive pulmonary disease (COPD), depression, anxiety, temperament, regulatory theory of temperament (RTT), mental health, psychosomatics, briskness, emotional reactivity, telemedicine, integrated care

## Abstract

**Introduction:** Asthma and chronic obstructive pulmonary disease (COPD) are chronic respiratory illnesses frequently accompanied by anxiety and depression. These psychological symptoms often go undetected due to their overlap with somatic complaints. According to the regulatory theory of temperament (RTT), biologically based temperament traits may influence emotional responses to chronic illness. This study examined whether RTT-defined temperament traits predict depression and anxiety severity in patients with asthma and/or COPD. **Material and Methods:** The study included 210 adult patients with asthma and/or COPD recruited from a university hospital and pulmonology clinics. Individuals with a prior history of mental illness were excluded. Participants completed three validated questionnaires: the Formal Characteristics of Behavior–Temperament Inventory (FCB-TI), the Beck Depression Inventory (BDI), and the State–Trait Anxiety Inventory (STAI). Additional demographic and clinical data were collected. Multiple linear regression was used to assess the predictive value of six temperament traits for depression, state anxiety, and trait anxiety. A significance threshold of α = 0.05 was used in all statistical tests. **Results:** Temperament structure significantly predicted all three mental health outcomes: depression (R^2^ = 0.37), state anxiety (R^2^ = 0.45), and trait anxiety (R^2^ = 0.35). Briskness negatively correlated with all outcomes, while emotional reactivity showed a positive correlation. No significant associations were found for the remaining four traits. Socioeconomic and lifestyle factors were not significant predictors. **Conclusions:** Temperament traits, particularly briskness and emotional reactivity, significantly influence depression and anxiety severity in asthma and COPD. Temperament assessment may serve as a low-cost, telemedicine-compatible tool to identify at-risk patients and support integrated, personalized care.

## 1. Introduction

The regulatory theory of temperament (RTT) emphasizes the role of temperament in the regulation of behavior, particularly in the context of stress. RTT defines temperament as a set of traits relating to formal aspects of behavior, such as emotional reactivity, briskness, activity, endurance, perseverance, and sensory sensitivity. Temperament is a component of personality that defines the properties of the central nervous system and can be observed in young children and animals. Studies of the correlation between temperament and somatic diseases show that depressiveness, hostility, and emotional reactivity are risk factors in the development of ischemic heart disease and lung cancer [1,2].

Anxiety is defined as an unpleasant emotional state characterized by feelings of anxiety, threat, and tension. Unlike fear, which is a response to a real, concrete threat, anxiety is a response to an anticipated, unspecified, or exaggerated threat. Anxiety tends to manifest at different intensity levels, ranging from mild anxiety to intense, paralyzing fear. Anxiety accompanies depression in up to 31.8% of the population [1].

Chronic obstructive pulmonary disease (COPD) is a respiratory disorder with a heterogeneous background characterized by chronic and recurring symptoms, such as dyspnea, excessive sputum production, cough, and recurrent exacerbations. The pathophysiology of COPD involves abnormalities in the airways and the alveoli, leading to a persistent and progressive airflow limitation, known as air trapping [3].

Asthma is an inflammatory respiratory syndrome. It can present with daily recurring symptoms, such as coughing, wheezing, chest tightness, and breathing difficulties, which affect the daily routine of patients. COPD is one of the few diseases in which morbidity, prevalence, and mortality are steadily increasing [4,5]. Asthma may also develop into COPD. The diagnosis of asthma and COPD necessitates strict adherence to medical advice, i.e., taking medication, follow-up appointments, attending rehabilitation classes, and following an appropriate diet, which, if not adhered to, can worsen somatic and psychological conditions [6]. An inverse relationship is also evident—reduced well-being or psychological problems experienced by patients can negatively affect adherence to medication, quality of life, number and length of hospitalisations, and even life expectancy [7,8,9]. Chronic diseases are often complicated by quality of life and mental problems. Somatic symptoms of depression and anxiety are nonspecific and can often be mistaken for symptoms of other diseases. They are also overrepresented in chronic respiratory diseases [10,11]. Depression and anxiety often limit one’s drive towards certain activities, while COPD and asthma limit the actual capabilities, in effect leading to the same effect of helplessness and apathy [12]. Therefore, the main problem with the swift diagnosis of psychiatric disorders in COPD and asthma is the fact that the symptoms presented by patients are often overlapping, making the outright diagnosis and differentiation nearly impossible [13].

In the current study, autoquestionnaires were used as a cost- and labour-effective tool for diagnosis and prevention of psychiatric comorbidities in chronic respiratory diseases. Often underappreciated by clinicists, psychological health is a major and factor in compliance and management of chronic diseases. Both anxiety and depression have a deleterious effect on treatment adherence, follow-up visits, and overall compliance, with a negative impact on prognosis and disease management.

Depression is the most common psychological disorder in the world [14,15]. It is estimated that 4–20% of the general population will exhibit symptoms of depressive disorders within their lifetime [16]. The prevalence of depression is significantly higher among people suffering from chronic respiratory diseases (7 to 42% in COPD) [17,18,19,20,21].

It is estimated that 18% of people worldwide live with asthma [22]. A significant proportion of cases are undiagnosed [23]. Undiagnosed people present with non-specific symptoms, such as coughing, wheezing, breathlessness, and, in severe exacerbations of asthma, visible work of the accessory respiratory muscles; however, because of the symptoms’ non-specificity, a diagnosis is not always made, and these people are not treated. According to some estimates, more than 300 million people are affected worldwide (7–10% of children, and about 5% of adults) [24], and more than 400,000 die annually. Asthma represents a major public health challenge, with the WHO estimating that more than 339 million people worldwide suffer from asthma [25]. Asthma is also the most common chronic respiratory disease in children, with an incidence of 0.6–15%. Based on age, we can distinguish early childhood asthma (≤5 years of age) and childhood asthma (>5 years of age) [26].

Despite its relatively low mortality rate, particularly in high-income countries, asthma ranks 16th among all diseases in terms of disability-adjusted life years (DALYs) lost [27]. Asthma is also a significant factor in the development of chronic obstructive pulmonary disease (COPD), which, in terms of deaths, is the fourth most common disease worldwide [28]. Primary prevention of asthma is currently not possible, as it would rely on genetic modifications or strict management of exposure to sensitizers in fetal life. Avoiding exposure to tobacco smoke in the womb and breastfeeding after birth reduces the risk of developing airway disease with wheezing as a symptom, but does not prevent asthma in later life. Currently, there are no recommendations for prenatal and postnatal allergen avoidance as a method of primary prevention. Secondary prevention is applicable in occupational asthma. Early cessation of exposure to noxious agents (allergens) in a sensitized person increases the chance of complete resolution of symptoms [23].

COPD is the fourth most common cause of death worldwide and is a major burden on health systems. In the USA, 5% of the population suffers from COPD [29], and worldwide, there are estimated to be up to 380 million patients [30,31,32]. It has become the most common cause of death in the USA and worldwide—after heart disease and cancer and before stroke [33]. Mortality from COPD is expected to rank third by 2030 [32,34]. The WHO 2024 report ranks it fourth in terms of mortality, after ischaemic heart disease, COVID-19 and stroke [35]. At the same time, in the context of the economic indicator of death rate in relation to R&D expenditure, COPD is the most underfunded of the diseases [36,37,38]. For comparison, in 2020, for every patient who died of asthma, $81,500 was spent on research and development, compared with only $800 for COPD. Given the relatively low investment in COPD treatment, one of the main methods of controlling COPD is behavioural–cognitive therapy, which is a relatively inexpensive and effective therapeutic option [39].

Early recognition of psychological components and reduction of their impact significantly improve therapeutic outcomes and quality of life indicators for patients [40]. It manages to limit costs and develop healthy expectations of progress in an incurable but treatable chronic condition. With properly implemented treatment, even incurable diseases, such as asthma and COPD, can be controlled to the extent that their negative impact on the patient’s quality of life is minimized. Proper adherence to treatment relies, however, on patients’ mental condition. It requires the patient to be ready, willing and able to follow the therapeutic instructions.

## 2. Materials and Methods

The main part of this prospective study was conducted among patients of the Norbert Barlicki Memorial Teaching Hospital No. 1 and specialized pulmonology outpatient clinics. A total of 210 volunteers were enrolled in the study based on their history of asthma and/or COPD. The main exclusion criterion was a history of mental disorders prior to the diagnosis of COPD and/or asthma, which ensured that the scope of the study was focused on mental disorders secondary to chronic respiratory disease. Patients who were qualified were invited to attend face-to-face meetings with the researcher during which they gave informed consent and completed a set of questionnaires. Each set of questionnaires required about 1.5 h to complete for each patient. The study design was evaluated and accepted by the Bioethics Committee at the Medical University of Lodz (administrative decision no RNN/308/18/KE, 15 March 2016).

Source data were obtained using three mental health assessment forms validated for the Polish population. The level of intensity of temperamental traits was determined using the Formal Behavioral Characteristics—Temperament Questionnaire (FCZ-KT) to obtain an individual profile of temperamental traits for each patient; (score correction was applied for the gender of the respondent). A depression symptom severity score and anxiety severity assessment were also obtained for each respondent using, respectively, the Beck Depression Inventory (BDI) and the STAI Self-Assessment Questionnaire—State and Trait Anxiety Inventory. All questionnaire were obtained from Pracownia Testów Psychologicznych Polskiego Towarzystwa Psychologicznego sp. z o.o., Warsaw, Poland.

Scores were obtained for BDI, STAI X1 and X2, and all 6 temperament traits as defined by RTT. The values obtained were analyzed using multiple linear regression (STATISTICA (data analysis software system), version 14.PL, www.statsoft.com).

### 2.1. Formal Characteristics of Behavior—Temperament Inventory (FCB-TI)

Regulatory theory of temperament, which focuses on formal aspects of behavior involving energetic characteristics, comprising traits, such as sensory sensitivity, emotional reactivity, endurance and activity and temporal characteristics—briskness and perseverance (Table 1).

The Formal Characteristics of Behavior—Temperament Inventory (FCB-TI) is a psychometric tool developed by Bogdan Zawadzki and Jan Strelau to assess basic temperament traits in adults and adolescents aged 15 years and older [41]. The theoretical basis of the questionnaire is the regulative theory of temperament (RTT) developed by Strelau, which was a continuation of Ivan Pavlov’s research on temperament. This work defines temperament as biologically determined personality traits that are relatively constant over time. The questionnaire is used to diagnose basic, biologically determined personality traits that describe formal aspects of behaviour.

The questionnaire is self-reporting in nature. It consists of 120 items, which are statements that require the patient to respond to each issue in the form of a ‘Yes’ or ‘No’ response. The items form the 6 basic temperamental scales.

### 2.2. Beck Depression Inventory (BDI)

The Beck Depression Scale (Beck Depression Inventory (BDI) [42,43] is a validated psychometric tool developed in the 1960s to assess the severity of depressive symptoms. This questionnaire is one of the most widely used tools in the world for the diagnosis of depression, both in clinical research and in medical practice. It is used to diagnose depression, monitor the emotional state of patients, and assess the effectiveness of therapeutic interventions in the course of the treatment.

The respondent responds by assessing their feelings within the past week [43]. As the patient completes the questionnaire independently, this allows the subjective severity of depressive symptoms to be assessed. In the structure of the BDI, each statement focuses on a different symptom of depression, which include sadness, pessimism, sense of failure, loss of pleasure, guilt, low self-esteem, self-punishment, suicidal thoughts, tearfulness, irritability, social withdrawal, inability to make decisions, dissatisfaction with self, change in body image, difficulties at work, sleep disturbance, fatigue, loss of appetite, weight loss, health concerns, and loss of interest in sex. Scores are totaled to give an overall score that classifies the level of depression from mild to severe.

### 2.3. State–Trait Anxiety Inventory (STAI)

The STAI questionnaire is a psychometric tool designed to measure two types of anxiety: state anxiety and trait anxiety. The STAI questionnaire consists of two subscales, each containing 20 items. The first subscale concerns the state of anxiety (S-Anxiety). It measures the temporary state of anxiety that the person is experiencing at any given time. The anxiety state items assess subjective feelings of tension, worry, nervousness, and arousal of the autonomic nervous system. The second subscale concerns the trait of anxiety (T-Anxiety). This scale assesses anxiety as a personality trait, i.e., a person’s tendency to react with anxiety to a variety of situations. The anxiety trait items explore the long-term predisposition to experience anxiety.

The STAI questionnaire is used in various fields, including clinical psychology, to diagnose and assess the severity of anxiety in patients. In biological research to study the relationship between anxiety and other variables, including psychological, social, and biological, and in medical practice to monitor anxiety in patients with somatic diseases, such as heart disease or cancer, and in the case of my study, asthma or COPD.

The STAI questionnaire is a tool with high reliability and validity, as confirmed by the numerous studies in which it has been used. Limitations of the STAI questionnaire include its subjectivity, through which results may be distorted by self-perceptions, and the inability of the questionnaire to differentiate between different types of anxiety, such as social anxiety or panic anxiety. The STAI questionnaire is a tool that offers a quick and effective way to understand both temporary and long-term aspects of anxiety in different contexts. It allows the diagnosis of anxiety with a distinction between anxiety as a state (temporary and situationally conditioned) and anxiety as a personality trait (a relatively fixed tendency).

A separate questionnaire was used to collect additional data that could support the interpretation of the obtained results. These supplementary questions addressed factors such as employment status, level of education, financial independence, comorbidities, and prescribed medications.

## 3. Results

Results of depression and anxiety inventory, personality traits assessment and socioeconomical data were obtained from 210 adult patients and volunteers and analyzed using multivariable linear regression. Multiple linear regression analyses were performed on three occasions. In each analysis, six temperament traits served as predictor variables, while the dependent variable differed across models: depression, anxiety as a trait, and anxiety as a state. The predictors were entered simultaneously using the enter method (single-step entry) to assess their collective contribution to the outcome variables. A significance threshold of α = 0.05 was used in all statistical tests.

### 3.1. Depression

Temperament trait structure is a significant predictor of depressive symptom severity. All temperament traits included explain 37% of the variance in the severity of depressive symptoms. Two temperament traits, that is, briskness and emotional reactivity, were found to be significant predictors of depression. The correlation between briskness and depression is negative, which means that as the intensity of this trait increases, the likelihood of developing depression in patients with asthma and COPD decreases. The relationship between emotional reactivity and depression is positive, which means that as the intensity of this trait increases, the likelihood of developing depression in patients with asthma and COPD increases (Table 2).

### 3.2. Anxiety (State)

The structure of temperamental traits is a significant predictor of anxiety severity, understood as a state. All temperament traits considered in this study explain 45% of the variance in anxiety severity as a state. Likewise, two temperamental traits, that is, briskness and emotional reactivity, were found to be significant predictors of anxiety as a state. The relationship between briskness and state anxiety is negative, which means that as the severity of this trait increases, the likelihood of developing anxiety symptoms in patients with asthma and COPD decreases. The correlation between emotional reactivity and state anxiety is positive; therefore, as the severity of state anxiety increases, the likelihood of developing anxiety symptoms in patients with asthma and COPD increases (Table 3).

### 3.3. Anxiety (Trait)

For anxiety considered as a trait, the structure of temperamental traits is a significant predictor of the severity of anxiety understood as a trait. All temperament traits analyzed explain 35% of the variance in anxiety severity as a trait. Briskness and emotional reactivity were found to be significant predictors of trait anxiety. The relationship between briskness and trait anxiety is negative, which means that as the intensity of this trait increases, the likelihood of developing a predisposition to experiencing anxiety in patients with asthma and COPD decreases. The relationship between emotional reactivity and trait anxiety is positive, which means that as the intensity of this trait increases, the predisposition to trait anxiety increases in patients with asthma and COPD (Table 4).

We have found no statistically significant correlations between any of the remaining four temperament traits and depression or anxiety. The supplementary questionnaire was used to screen for additional factors that might be relevant; however, commonly cited risk factors, such as poor living conditions and smoking habits, failed to provide statistically significant results.

## 4. Discussion

In this study, we have attempted to explore correlations between temperament traits and susceptibility to anxiety and depression developed secondary to asthma and COPD, the key point being a causal relation and mental disorders being a consequence of chronic illness. Understanding the driving force behind mental disorders can aid in their alleviation and boost compliance in dealing with somatic diseases by limiting the development of complications. Early identification of patients at risk will enable the prevention of mental disorder development or, at the very least, facilitate the earliest possible therapeutic intervention in the form of targeted therapy and an individualized approach to patients, all this with relatively low cost and high accuracy. Similar approaches are explored in the context of multiple other chronic diseases; however, they usually focus on correlation without exploring causality. In our study, we have shown that high briskness and low emotional reactivity constitute protective factors in the context of secondary depression and anxiety. An analytic and adaptable temperament is a key to withstanding hardships without suffering overwhelming emotional burdens in the form of stress and depression. Upon recognizing these two factors in a patient, the attending physician should strongly consider facilitation of a consult with a mental health specialist to better prepare the patient for the challenges of living with a chronic disease and managing expectations. Establishing a realistic outlook from the very beginning of a chronic disease provides more realistic expectations that are easier to meet and manage. Hopefully, this approach will prove universally viable for chronic diseases other than asthma and COPD. Screening patients based on their temperamental traits can be helpful in differential diagnosis, since symptoms presented in asthma, COPD, anxiety and depression are often overlapping, and this symptom-independent diagnostic tool will prove helpful in differentiating the source of the problem.

The temperament can be described using several different tools, each using different categories and classifications of traits. Although they are describing a common subject, using different measuring tools leads to results that are not clearly translatable and comparable. The most used tools for describing temperament are the temperament and character inventory, the regulative theory of temperament, and the Memphis, Pisa, Paris, and San Diego Autoquestionnaire.

Studies relying on RTT as a temperament assessment tool were conducted on HIV/AIDS [44] and rheumatoid arthritis [45] patients. For HIV, the relation was positive for emotional reactivity and inversely proportional for briskness, sensory sensitivity, and activity. For AIDS, a positive correlation was found with emotional reactivity and a negative one with sensory sensitivity. These were consistent with our results, indicating an inversely proportional relationship between briskness and depression and anxiety, and simultaneously, the risk of developing these mental disorders was directly proportional to the intensity of the emotional reactivity of patients.

Cloninger’s TCI was used to describe temperament in studies on tinnitus [46], chronic pain [47], urticaria [48] endometriosis [49] and burning mouth syndrome [50]; the results consistently highlighted that development of depression and anxiety is correlated with higher harm avoidance and lower self-directedness (novelty seeking, persistence, reward dependence).

Studies using the Memphis, Pisa, Paris, and San Diego Autoquestionnaire were conducted on fibromyalgia [51] and type 2 diabetes [52]. Both studies equivocally pointed towards optimistic, hyperthymic temperament as a protective factor in the development of depression and anxiety.

Although the results obtained in above-mentioned studies cannot be directly compared due to differences in definitions of temperamental traits and dimensions, all these types of personality point towards resistance to depression and anxiety fortified by high briskness and vulnerability associated with high emotional reactivity. The main advantage of our study, however, is the fact that we have managed to distill the positive and negative temperament traits to just those two factors and, simultaneously, to prove the causality, not a mere coexistence, between psychiatric and somatic problems.

Cognitive behavioral therapy can help patients understand and manage their reactions to stressful events. Understanding emotions increases the effectiveness of the therapy and improves doctor–patient communication, which is why psychologists should be involved in managing chronic disease [53]. Profiling the temperament can prove beneficial in the prediction of risk factors and disease resistance.

Our data do not support the common thesis that socioeconomical factors such as standard of living, employment and education profile correlate with increased risk of developing psychiatric disorders, but we must admit that this can largely be attributed to highly diverse population sample, which resulted in many categories that cannot be reliably analyzed with statistical significance. This can, however, be rectified by the continuous collection of data from new and returning patients.

Another limitation of our study results from the self-assessment nature of the tools used. This is a double-edged sword, which, on one hand, provides a subjective point of view that is greatly relevant for psychiatric profiling, but fails to provide objective clinical data. We have, however, judged this tradeoff worthwhile, since one of our main focuses was on the universal applicability of our research. Segmentation of our sample group based on the severity of disease would lead to an unreliably low sample size.

## 5. Conclusions

In conclusion, identifying potential psychological risk factors enables a more tailored approach to personalized medicine. The proposed assessment of temperament can be effectively implemented through telemedicine, helping to reduce stigma, enhance accessibility, and promote an integrated model of care that unites psychiatric and pulmonological support. Such a broad, multidisciplinary approach is more efficient and cost-effective than narrowly focusing on somatic factors alone. Patients at risk of developing mental disorders secondary to chronic respiratory disease can be proactively connected to psychological care and support groups, reducing the likelihood and severity of anxiety and depression. This, in turn, significantly enhances both quality of life and long-term prognosis.

## Figures and Tables

**Table 1:** Temperament traits according to the regulative theory of temperament.

Temporal Characteristic of Temperament	
Briskness	Briskness is a tendency of an individual that favours a quick reaction, maintaining a high tempo of activities and an ease of shifting reactions in response to dynamically changing environmental factors.
Perseverance	Perseverance can be defined by recurrence and persistence of actions; it is a somewhat stubborn tendency to continue and/or repeat behavior even after cessation of the stimuli, which evoked given behavior in the first place
**Energetic Characteristics of Temperament**	
Sensory Sensitivity	Sensory sensitivity is defined as the ability to all manners of sensory stimuli. High sensitivity allows for reaction to signals of low stimulatory value.
Endurance	Endurance is defined by personal endurance against distractors and fatigue; it is an ability to maintain adequate reactions in situations demanding long-lasting and high-stimulative activity
Emotional Reactivity	Emotional reactivity is composed of emotional sensitivity and emotional endurance; it describes a tendency to react to emotion-generating stimuli.
Activity	Personal activity is defined by direct and indirect sources of stimulation; it is a tendency to partake in highly stimulative behaviours or to seek external stimulation through one’s behaviour.

**Table 2:** Analysis of temperament traits in depression.

Predictors	*β*	*t*	*p*
Briskness	**−0.345**	**−4.541**	**<0.001**
Perseverance	−0.148	−2.069	0.060
Sensory sensitivity	−0.082	−1.372	0.172
Emotional reactivity	**0.417**	**4.821**	**<0.001**
Endurance	0.027	0.340	0.734
Activity	−0.035	−0.530	0.597

Note: *β* = standardized regression coefficient; *t* = *t*-value for the predictor’s significance test; *p* = probability value indicating statistical significance. Model summary: *F*(6, 201) = 21.33, *p* < 0.001, adjusted *R*^2^ = 0.37, statistically significant results in **bold**.

**Table 3:** Analysis of temperament traits in anxiety (state).

Predictors	*β*	*t*	*p*
Briskness	**−0.250**	**−3.263**	**0.001**
Perseverance	−0.018	−0.246	0.806
Sensory sensitivity	0.045	0.747	0.456
Emotional reactivity	**0.461**	**5.275**	**<0.001**
Endurance	0.042	0.522	0.602
Activity	−0.050	−0.741	0.460

Note: *β* = standardized regression coefficient; *t* = *t*-value for the predictor’s significance test; *p* = probability value indicating statistical significance. Model summary: *F*(6, 203) = 20,12, *p* < 0.001, adjusted *R*^2^ = 0.35, statistically significant results in **bold**.

**Table 4:** Analysis of temperament traits in anxiety (trait).

Predictors	*β*	*t*	*p*
Briskness	**−0.209**	**−2.945**	**0.004**
Perseverance	0.006	0.082	0.935
Sensory sensitivity	−0.031	−0.553	0.581
Emotional reactivity	**0.493**	**6.097**	**<0.001**
Endurance	−0.066	−0.901	0.369
Activity	0.019	0.312	0.755

Note. *β* = standardized regression coefficient; *t* = *t*-value for the predictor’s significance test; *p* = probability value indicating statistical significance. Statistical Model summary: *F*(6, 203) = 29.97, *p* < 0.001, adjusted *R*^2^ = 0.45, statistically significant results in **bold**.

## Data Availability

The data presented in this study are available on request from the corresponding author due to copyright status and intellectual property protection on used questionnaires as well as General Data Protection Regulation since all items were personally signed by participants.

## Abbreviations

The following abbreviations are used in this manuscript:

TCI	Temperament and Character Inventory
RTT	Regulative Theory of Temperament
STAI	State-Trait Anxiety Inventory
COPD	Chronic Obstructive Pulmonary Disease
